# Globalization and social determinants of health: Promoting health equity in global governance (part 3 of 3)

**DOI:** 10.1186/1744-8603-3-7

**Published:** 2007-06-19

**Authors:** Ronald Labonté, Ted Schrecker

**Affiliations:** 1 Department of Epidemiology and Community Medicine, Faculty of Medicine and Institute of Population Health, University of Ottawa, Canada; 2 Department of Epidemiology and Community Medicine, Faculty of Medicine and Institute of Population Health, University of Ottawa, Canada

## Abstract

This article is the third in a three-part review of research on globalization and the social determinants of health (SDH). In the first article of the series, we identified and defended an economically oriented definition of globalization and addressed a number of important conceptual and metholodogical issues. In the second article, we identified and described seven key clusters of pathways relevant to globalization's influence on SDH. This discussion provided the basis for the premise from which we begin this article: interventions to reduce health inequities by way of SDH are inextricably linked with social protection, economic management and development strategy.

Reflecting this insight, and against the background of the Millennium Development Goals (MDGs), we focus on the asymmetrical distribution of gains, losses and power that is characteristic of globalization in its current form and identify a number of areas for innovation on the part of the international community: making more resources available for health systems, as part of the more general task of expanding and improving development assistance; expanding debt relief and taking poverty reduction more seriously; reforming the international trade regime; considering the implications of health as a human right; and protecting the policy space available to national governments to address social determinants of health, notably with respect to the hypermobility of financial capital. We conclude by suggesting that responses to globalization's effects on social determinants of health can be classified with reference to two contrasting visions of the future, reflecting quite distinct values.

## Background

This article is the third in a three-part review of research on globalization and the social determinants of health (SDH). In the first article of the series, we identified and defended an economically oriented definition of globalization and addressed a number of important conceptual and methodological issues. In the second article, we identified and described seven key clusters of pathways relevant to globalization's influence on SDH. This discussion provided the basis for the premise from which we begin this article: interventions to reduce health inequities by way of SDH are inextricably linked with social protection policy, economic management and development strategy.

It follows that when the objective is to reduce health inequities by way of SDH, the scale at which an intervention must be implemented is not necessarily the scale at which the problem arises. For example, addressing the poverty of individuals and households may demand policy responses on the part of state/provincial and national governments, yet they may be limited in their ability to act effectively because of constraints that are created by, and can best be changed by, actors outside their national borders, such as multilateral institutions or institutional investors. This interconnectedness is a distinguishing characteristic of contemporary globalization, and provides the basis for Pogge's argument that the industrialized world has an ethical obligation to reduce poverty outside its own borders [1]. We do not mean here to write domestic political action out of the picture; far from it. Szreter's work on industrializing England shows that the formation of effective domestic political coalitions was necessary to the translation of economic growth into improved population health status [2-4]. However globalization shapes the environment within which such coalitions operate, and affects their chances of success in a variety of ways.

In 2000, a resolution of the UN General Assembly committed the international community to achieving the Millennium Development Goals (MDGs), by the year 2015 in most cases. Three of the Goals, which involve reducing child and maternal mortality and reversing the spread of HIV/AIDS, malaria, and other communicable diseases, are explicitly health-related. Four others directly address crucial social determinants of (ill) health: extreme poverty, undernourishment, environmental hazards, and lack of access to education. Targets that have been developed with respect to each of the goals state more specific milestones, such as reducing by half the proportion of the world's people without safe drinking water [5]

The MDGs arguably represent a 'first' in terms of commitments by the international community to a specific development agenda. They are unambitious when viewed against the sheer volume of unmet basic human needs. Particularly notable is the modesty of the poverty reduction target (reducing by half, in the year 2015, the proportion of the world's people living on less than $1/day) when viewed against the background of expanding global affluence [6]. Similarly, compare the MDG 7 target of improving the lives of 100 million slum dwellers per year by 2020 with the estimate that if present trends continue, 1.4 billion people worldwide will live in slums in 2020 [7]. A further problem is that, apart from MDG 3 on gender equity in education, the MDGs are stated in terms of societal averages – meaning that a country may be able to achieve MDG targets related to health, such as under-5 mortality, while failing to improve the health status of the worst-off groups [8,9].

On the other hand, the MDGs are ambitious when viewed against the uneven pace of recent progress toward meeting the needs they address. Substantial progress has been made toward achieving the MDG targets in some regions. In others, especially sub-Saharan Africa, the situation is grim [10,11]. Recent syntheses of available evidence, notably those by the UK Commission on Africa and the UN Millennium Project, describe an emerging consensus that if the MDG targets or comparable improvements in human well being are to be achieved, then substantial long-term commitments of additional resources by the industrialized countries are necessary [12,13]; see also [14](p. 190–192). Because an increasingly dense network of trade and investment flows links rich and poor across national borders, achieving the MDGs or comparable goals will also require revamping the trade and foreign policies of the industrialized world to ensure compatibility with progress toward the MDGs and other objectives related to basic needs, and to address the "asymmetrical" distribution of gains, losses and power that is characteristic of globalization in its current form, [15,16] as noted in the second article of the series.

Several elements of that asymmetry are directly relevant to issues of global governance. In the case of trade policy, for instance, many developing countries cannot afford the professional expertise that is needed to participate effectively in multiple trade negotiations and to pursue dispute resolution [17] – creating a strong case based on fairness for expanded assistance in capacity building. It is more difficult to get around the asymmetry created by differences in market size as they affect not only initial bargaining positions but also the ability to make use of dispute resolution even when the outcome is favourable. "The sanction for violating a WTO agreement is the imposition of duties. If Ecuador, say, were to impose a duty on goods that it imports from the United States, it would have a negligible effect on the American producer; while if the United States were to impose a duty on goods produced by Ecuador, the economic impact is more likely to be devastating" [18](p. 504).

There follows a generic overview of key policy imperatives and opportunities. It is incomplete in at least three respects. First, it focuses on policy actions at the international level, rather than on mitigative or compensatory policies that can be adopted at the national or subnational level, apart from a discussion of the extent to which the international economic and political context creates constraints that limit the ability of governments to adopt such policies. Second, it does not address some important governance issues raised by changing distribution of power and economic resources outside the industrialized world – exemplified by the rise of China and, to a lesser extent, India as global economic players [19] and by the emergence of world-scale resource corporations like Brazilian-based Companhia Vale do Rio Doce, which recently acquired Canadian mining giant Inco Ltd. Third, it focuses on eliminating current barriers and constraints rather than on opportunities associated with the potential emergence of new forms and institutions of global governance. Those opportunities represent an important area for building research collaborations and communities of practice that link development policy, clinical disciplines, population health and social science fields such as international relations and political economy.

### Making more resources available for equitable access to health systems

Health care and health systems are among the SDH, and an immediate imperative is to make more resources available to deliver key interventions. The Commission on Macroeconomics and Health estimated in 2001 that routinely providing a package of basic, relatively well understood low-cost and low-tech interventions [20], costing US $34 per capita per year and comprising "a rather minimal health system," could save "at least 8 million lives *each year *by the end of this decade" [21](emphasis in original). This figure must be compared with average national health expenditure of $24 per capita in 2001 in jurisdictions that the World Bank defines as low income countries, where 2.2 billion people live. In countries in which half of those people live – more than a billion, in other words – annual per capita spending on health was $14 per capita or less, according to the World Bank's Health, Nutrition and Population (HNP) database [22](accessed May 9, 2006). Not all of this expenditure, of course, involves services for the poor or otherwise vulnerable, and not all of it is public spending: recall the pervasiveness of "medical poverty traps" noted in the second article of this series and consider that in Viet Nam, a country where poverty induced by catastrophic illness is a major problem [23], *public *health care expenditure stood at less than $4 per capita in 2001 [24] – reflecting a general and ironic trend for private, out-of-pocket payment to comprise a high proportion of total health expenditure in many of the world's poorest countries.

The Commission on Macroeconomics and Health and, more recently, the Commission for Africa and the UN Millennium Project all argued strongly for a several-fold increase in the value of development assistance for health, focused on basic interventions. The Commission for Africa [12](p. 196) was also explicit in recommending that elimination of user fees be supported by long-term donor financing commitments – essential if the increased use of services that follows the elimination of financial barriers is not to create demands that already overstressed public health systems cannot meet. The need for such commitments underscores the fact that many low-income countries will require substantial development assistance for many years, probably for decades, if their health systems are to be financed at the minimum level identified by the Commission on Macroeconomics and Health [25,26]. The urgency of providing such additional resources is clear and should not require further elaboration, but one argument is worth citing. Economist Jeffrey Sachs, who chaired both the Commission on Macroeconomics and Health and the more recent Millennium Project, has estimated [27] that the combination of low per capita income and weak government institutions in many tropical sub-Saharan African countries might be capable of generating US$50/capita in total public revenue. "This tiny sum must be divided among all government functions ... [T]he health sector is lucky to claim $10 per person per year out of this, but even rudimentary health care requires roughly four times that amount .... Foreign aid is therefore not a luxury for African health. It is a life-and-death necessity."

However, rich countries have so far not even lived up to the rhetoric associated with their highest profile initiative to increase support for health in the developing world. The Global Fund to Fight AIDS, Tuberculosis and Malaria was hailed at the 2001 G8 Summit as a "a quantum leap in the fight against infectious diseases," yet the Fund continues to lack a long-term financing mechanism. It relies instead on periodic replenishment meetings that in effect involve passing a hat, and has estimated that it will need $7.1 billion in 2006 and 2007 to fund new proposals and continuations of existing work [28]. The September 2005 replenishment meeting raised the total value of funds pledged for 2006–2007 to $3.73 billion, or just over half the anticipated funding requirement for those years [29]. This creates serious constraints on what activities the Fund can support even after scientific merit has been demonstrated, since the Fund "can only approve grants if the full amount required for the first two years is covered by pledges from donors in the calendar year of the approval" [28](p. 34). The Fund itself now estimates that future funding requirements could be as high as $7–8 billion per year [28](p. 32), and a stable source of long-term financing, such as a global trust fund, is still not in place.

Provision of public goods related to health presents distinctive problems. In common usage, the phrase "public good" is often associated with the common welfare, or with such values as equity and social justice. Its definition in economic theory is more precise: a *private good *(either a service or a good in the physical sense) is one whose individual consumption is both excludable (my use of the good is not dependent on others' use) and rivalrous (my use of the good could preclude use by another). Conversely, a *public good *is one that is non-excludable (classic illustrations are the order created by traffic lights and, from the days before GPS, the safety benefits of lighthouses) and, in pure form, is non-rivalrous (my use of the traffic light, lighthouse or GPS signal in no way impairs your use of it). Few pure public goods exist and public policy choices, which may vary over time, often determine the balance between private and public characteristics of a good [30,31]. Although health itself is not a public good, numerous public goods *for *health exist, including scientific knowledge and communicable disease control. The terminology of global public goods for health (GPGH) is now in widespread use, but a recent WHO research initiative [32] concluded that many public goods for health are in fact regional, rather than global. Malaria control is a case in point [33](p. 23); since malaria is primarily a disease of poor regions, this fact may account for the serious underfunding of or attention to malaria control on the part of the industrialized world [34].

Whether global or regional, many public goods for health, such as communicable disease control (including vaccination) and control of antibiotic resistance, are conspicuously undersupplied in the marketplace, reflecting the "dramatic decay in local and global public health capacity" identified by the United Nations High Level Panel on Threats, Challenges and Change [35]. In theory scientific knowledge is a quintessential public good, yet in practice it is often ring-fenced by mechanisms such as intellectual property rights. This is arguably both cause and consequence of increased reliance on private financing of health research: in 2001, private for-profit companies spent $51.2 billion on health research, as against $46.6 billion in public spending [36](p. x), but as noted in the second article of this series priorities for privately financed research are more likely to be shaped by anticipated profit than by contribution to reducing the global burden of disease. A further complication arises from the fact that potential for commercialization is an increasingly important consideration for at least some national, publicly financed health research granting agencies. Commercially oriented research priorities are likely entirely to ignore interventions both within and outside the health sector that address disparities in SDH, since such interventions are intrinsically not amenable to commercialization. Thus, it is imperative to develop new mechanisms for financing health research that do not rely on the anticipation of profit and avoid the resulting skewing of priorities; reform of national and international intellectual property regimes is arguably a part of such necessary reforms [37-39], but just a part (see e.g. [40]).

### Expanding and improving development assistance

The need for more resources for health systems and to support provision of health-related public goods is just one argument among many for increasing the value of official development assistance (ODA). ODA is the most visible and conspicuous transfer of resources from rich to poor countries, although it is far from being the single largest contributor to international financial flows. The UN Millennium Project and the UK Commission for Africa each concluded that an approximate doubling of current ODA spending is necessary, although not sufficient, if much of the developing world is to have a chance of achieving the MDGs [12,13]. The Millennium Project report was also noteworthy for recommending major changes in how ODA spending is directed in order to increase its relevance to the MDGs, thereby lending support to long-standing criticisms of aid agencies for providing assistance for specific projects rather than as general budget support and for the multiple reporting requirements they demand of recipients [13](p. 193–210). At their 2005 Summit, the G8 countries committed themselves to an additional $25 billion in development assistance to Africa by 2010; this commitment can be read as a direct response to the report of the Commission of Africa, which was part of the British Prime Minister's initiative to situate African development as one of the main items on the Summit's agenda. It remains to be seen how effectively the G8 will live up to the Gleneagles aid commitments, and whether the increase will come at the expense of aid flows to other regions of the world, where national-level statistical indicators may be less bleak but poverty and other deficiencies in access to SDH are nevertheless widespread.

Some commentators were and are sceptical about the value of these commitments for a different reason. They argue that domestic governance failures, capacity limitations, and the tendency of African countries in particular toward "neopatrimonial systems of rule" [41] will render such inflows ineffective if not destructive [42,43]. The Commission for Africa and the Millennium Project each examined the evidence and made numerous recommendations for improving the effectiveness with which aid is used to achieve the MDGs and similar objectives, which cannot be reviewed in this series. More importantly though, each initiative directly challenged fashionable scepticism about the value of development assistance, crucially emphasizing *donor *policies and practices as constraints on aid effectiveness. The Millennium Project report further pointed out the irony that "the notion of taking the [Millennium Development] Goals seriously remains highly unorthodox among development practitioners" *because of *a lack of financial support from the industrialized world [13](p. 202). In a direct rejection of received wisdom that weak governance or "absorptive capacity" constraints seriously limit the potential benefits from short-term increases in development assistance, its discussion of Africa concluded that the quality of governance in African countries is comparable to that in other regions with similarly low incomes, noting that "good governance requires resources for wages, training, information systems, and so forth" [13](p. 146).

Important changes in delivery mechanisms and funding criteria to improve the effectiveness of aid in contributing to health equity can and should be made (see e.g. [44]). However, it is to be hoped that the Millennium Project and the Commission for Africa have decisively shifted the burden of proof to those resisting substantial new ODA commitments to show how meaningful improvements in health equity and access to SDH can be achieved in the absence of such commitments, and to the rich countries to demonstrate mechanisms for making the necessary resources available without compromising their effectiveness through ties to their own economic and strategic interests.

### Expanding debt relief and taking poverty reduction (more) seriously

**Figure 1:**
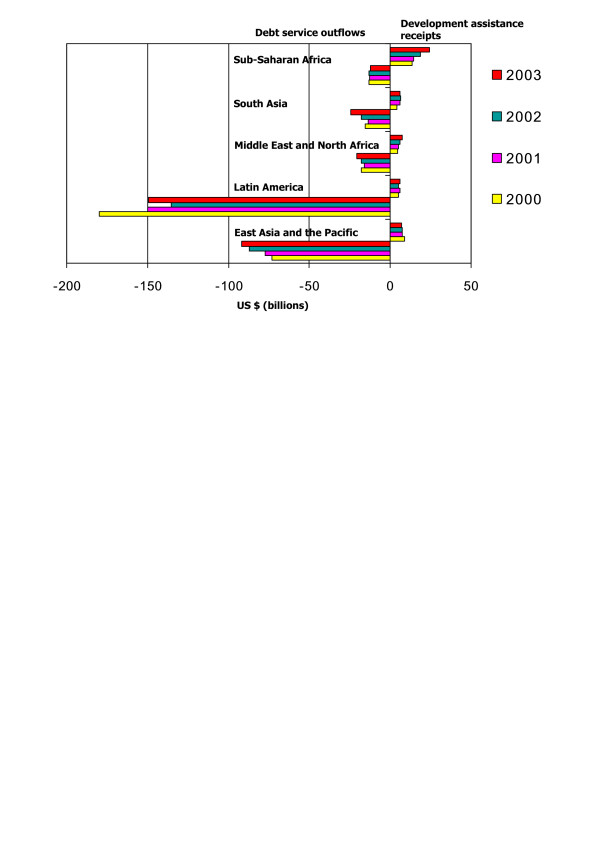
Debt service and development assistance, by region, 2000–2003. Source: World Bank Data from Econstats  and ; accessed February, 2007).

External debt remains perhaps the most serious constraint on aid's effectiveness: " [D]ozens of heavily indebted poor and middle-income countries are forced by creditor governments to spend large parts of their limited tax receipts on debt service, undermining their ability to finance investments in human capital and infrastructure. In a pointless and debilitating churning of resources, the creditors provide development assistance with one hand and then withdraw it in debt servicing with the other" [13](p. 35). In every region of the developing world except sub-Saharan Africa, inflows of development assistance are more than offset by the annual outflow of debt service payments to external creditors (Figure 1), and in sub-Saharan Africa the high percentage of many government budgets accounted for by development assistance means that *any *drain on scarce financial resource to service external debt represents a serious constraint. Over the last ten years, the rich countries have offered gradually increasing levels of debt cancellation to a limited number of the world's poorest countries through the Heavily Indebted Poor Countries (HIPC) initiative. Although debt cancellation for HIPCs has made possible increases in public spending on such basic needs as health and education in several recipient countries [45], many HIPCs have seen only modest decreases in their debt service obligations, and three have actually seen *increases *[46](p. 148). In addition, the eligible countries are not those where a majority of the world's poor people live: many other countries are not statistically desperate enough to qualify, despite high levels of poverty and high external debt burden [47,48]. Both limitations arise from the fact that a "sustainable" debt load has been defined for purposes of the HIPC initiative with reference to a ratio of debt service to annual export revenues, based on what have often turned out to be optimistic projections of export earnings and commodity prices.

This debt sustainability criterion was adopted at the insistence of the G7, "balancing the need to include strategic G7 allies and the desire to help keep costs down" [49](p. 17–18). Various refinements of this criterion are now under consideration [46](p. 152–154), but none explicitly incorporates the alternative principle of working backward from the value of government expenditure required to meet basic needs, and only then determining how much (if any) of the public budget can be devoted to debt repayment [50-52]. The Millennium Project echoed many earlier critiques in recommending that: "'Debt sustainability' should be redefined as 'the level of debt consistent with achieving the Millennium Development Goals,' arriving in 2015 without a new debt overhang. For many heavily indebted poor countries this will require 100 percent debt cancellation. For many heavily indebted middle-income countries this will require more debt relief than has been on offer" [13](p. 207–208). Thus, expanding both the availability of debt relief and its value must be a priority from the standpoint of health equity and SDH.

At the 2005 Summit, the G8 committed themselves to increasing the value of debt relief by cancelling all debts owed by HIPCs to the World Bank, IMF and the concessional (i.e., low-interest) arm of the African Development Bank once the relevant countries reach the HIPC completion point. "To reach completion point, countries must maintain macroeconomic stability under a PRGF-supported program, carry out key structural and social reforms, and implement a Poverty Reduction Strategy satisfactorily for one year," after which debt relief is provided by the creditors that have signed up for the HIPC initiative [53](accessed May 13, 2006; PRGF is the Poverty Reduction and Growth Facility, formerly the Enhanced Structural Adjustment Facility, of the IMF). The 2005 Summit commitment, now formalized as the Multilateral Debt Relief Initiative (MDRI), was a welcome next step, as was a separate partial debt cancellation deal for Nigeria estimated to be worth $31 billion [54]. However, the reliability of the MDRI commitment is called into question by the fact that as of mid-2005, existing (i.e. pre-Summit) debt cancellation commitments under HIPC were underfunded by approximately $12.3 billion, and were facing non-participation by many commercial creditors [46](p. 146) Further, additional debt relief under MDRI will be at least partly offset by reductions in development assistance [55], thus repeating the shell game in which development assistance declined substantially in the late 1990s after the start of the original HIPC initiative [56](p. 6–7). Indeed, because development assistance spending for 2005 includes major one-off debt cancellations for Iraq and Nigeria, without further new commitments (which have not yet been forthcoming), overall ODA spending may actually decline in 2006, for which data are not yet available, and 2007 [57].

As noted in the second article of the series, in order to qualify for debt relief under HIPC, national governments have had to prepare Poverty Reduction Strategy Papers (PRSPs) for approval by the World Bank and IMF, and to update them periodically. Although the process offers great potential benefit, in practice direct parallels exist between the PRSP process of qualifying for debt relief and earlier forms of conditionality [58,59]; recent studies confirm the continuity of the macroeconomic principles embodied in PRSPs with the earlier era of structural adjustment [60-62]. For example, PRSPs may include "trade-related conditions that are more stringent, in terms of requiring more, or faster, or deeper liberalization, than WTO provisions to which the respective country has agreed" [63](p. 20). Even if one rejects the position that PRSPs are being used quite cynically as a vehicle to pry open developing country markets, it appears that the lending institutions that demand and assess PRSPs continue to operate on the uncritical presumption that development is best achieved through rapid integration into the global economy, without consideration of economic distribution or health equity impacts.

Further questions about the architecture of development assistance and debt relief involve effects on public health and education budgets of the expenditure ceilings on which the IMF, in particular, is reported to insist as elements of PRSPs and macroeconomic management plans, even when the necessary resources have been committed by external donors. The economic rationale involves limiting inflation and currency appreciation, with the latter viewed with special concern because it could reduce the competitiveness of a country's exports and hence its ability to repay external debts. A 2005 article based on previous research and the field experience of one of the authors identified this constraint as operating in a number of countries including Mozambique, Tanzania and Uganda [64,65]. In response, World Bank and IMF officials argued that Medium-Term Expenditure Frameworks (MTEFs) incorporating public sector expenditure ceilings "are not a reflection of some malign intent," but rather "state what money is available and what programmes are possible within the context of that resource envelope" [66]. They provided no country-specific evidence to counter the argument that such expenditure ceilings are compromising national governments' ability to meet basic needs. Subsequent analyses [65,67] have strengthened the case against expenditure ceilings. A full assessment is difficult given the lack of transparency and the asymmetrical nature of relations between the IMF and national governments. Nevertheless, it is clear that the IMF approach does not reflect a willingness to revisit past policy choices and address present-day asymmetries in resources and bargaining power that together determine "what money is available" to a particular society or national government: say, one in sub-Saharan Africa trying to deal simultaneously with declining commodity prices, the impact of the HIV-AIDS epidemic, and the legacy of capital flight facilitated by hospitable financial centres in the developed world.

Finally, it is important to challenge the legitimacy of external creditors' financial claims when they involve repayment of funds lent to governments that systematically looted the public treasury or used public funds (including those supplied by external borrowers) for domestic repression in order to maintain power. Pogge [68] questions the legitimacy of these debts on ethical grounds, since the international community need not have permitted violently repressive or larcenous rulers to borrow against the assets and future earnings of their subjects, which is what they did in many cases. Other commentators have similarly questioned whether "odious debts" are collectible as a matter of international law [69,70]. The international community remains obstinate in its failure to confront this question, which needs to be explored with special urgency in cases where the imperative of repaying external creditors threatens to conflict with domestic public expenditure priorities related to health equity and SDH. In our view, and that of other commentators on debt issues [47,52,71,72], the latter must always take priority, and the onus is now on the industrialized countries individually and collectively to develop concrete policy responses.

### Making trade policy development-friendly

Something close to a new conventional wisdom has grown up around the relation between trade and development. Organizations otherwise as divergent in their perspectives as Oxfam and the World Bank apparently agree on the value to developing economies, especially the world's poorest countries, of access to industrialized world markets – sometimes citing figures to the effect that annual gains from complete liberalization of trade would amount to several times the value of development assistance [73,74]. Because markets for agricultural commodities are economically critical for many developing countries agricultural subsidies, which simultaneously lower prices within the borders of the producing country and enable producers to export at artificially low prices, are a special concern. So, too, is the continuing use of tariff escalation on high value-added or manufactured exports from poorer nations by industrialized countries, in contrast to low or zero tariffs on raw commodity exports. Improved access to developed country markets for manufactured products could yield very substantial income gains for the developing world [75,76], although estimating the value of potential markets lost to developing country producers as a result of subsidies and trade restrictions is fraught with difficulty [77].

Birdsall and colleagues [78] question the new conventional wisdom. They argue, unfortunately without supporting documentation, that the effects of agricultural subsidies on international prices of commodities such as cotton are far too small to affect the competitiveness of developing country producers in their own or export markets. While reserving judgment on this argument, it must be acknowledged that the relations between agricultural subsidies as defined and prospects for development are more complicated than acknowledged by many participants in the debates [77,79,80]. Although improved market access may increase the incomes of developing country agricultural producers who are already part of the cash economy, it is likely to have little benefit for larger numbers of producers who are primarily oriented toward subsistence, with occasional local market sales – the problem of "two agricultures" [80]; see also [81]. The entire issue of agricultural trade and SDH requires "a more fine-grained approach, which would differentiate among crops and countries" [82](p. 45). In the aftermath of the collapse of WTO negotiations in July, 2006 because of failure to make progress on agricultural subsidies, that prospect is perhaps more remote than ever.

Apart from the specifics of agricultural trade, multiple ironies surround the relation between contemporary trade policy priorities and the ability of developing countries to meet basic needs related to SDH. At a theoretical level, "the arguments advanced in favour of trade liberalization as a way of facilitating learning and productivity growth call for support and protection in the early stages of large scale, specialized enterprises, not full exposure of them to foreign competition" [75](p. 10). This strategy was adopted, with variations, by countries such as China, Korea and Vietnam that are now held up as exemplars of the benefits of globalization: they opened up their markets to imports selectively as their previously protected industries matured, and adopted intellectual property regimes that favoured domestic producers, just as European and North American countries had done a century earlier [78,83,84]. Not only current bilateral and multilateral trade agreements but also informal pressure from the industrialized world may now preclude similar development strategies by later industrializers [85,86]: the reason economist Ha-Joon Chang refers to the trade policy stance adopted by the industrialized countries as "kicking away the ladder."

Two examples suffice to show the importance of this dynamic for development – and thus, by implication, for SDH. First, as noted earlier PRSPs have been used as a source of leverage for import liberalization, without considering impact on countries' ability to meet basic needs related to health. Second, provisions for Special and Differential Treatment (SDT) have been a feature of the world trading regime since the early postwar years; they embody recognition of the distinctive needs of countries at vastly different stages of economic development. However the SDT provisions in the General Agreement on Tariffs and Trade (GATT) were seriously weakened, in terms of their value for developing economies, with the advent of the WTO. Intense lobbying by African and Asian countries led to a commitment by WTO members in 2001 to review "all Special and Differential provisions...with a view to *strengthening *them and making them more precise, effective and operational" [87](¶44, emphasis added). But what should count as strengthening? The fundamental question is whether SDT provisions should be considered temporary measures to facilitate the integration of developing economies into today's trade policy regime, or whether "the bottom-line question for the WTO should be what it can do to facilitate development, not what it is willing to allow to ease adjustment" [88](p. 300). This issue remains unresolved, and arises even more acutely with respect to the proliferation of bilateral and regional trade negotiations and agreements [89](pp. 27–56). In such negotiations and relationships, disparities in bargaining power and resources may be even more glaring than at the WTO. As a result, "WTO-plus" provisions emerging from these settings may vitiate whatever gains in terms of market access and domestic policy flexibility that developing countries are able to secure within the WTO framework [90]. This is a special concern given the likelihood that bilateral and regional negotiations will become even more important following the events of July 2006.

### Treating health as a human right: What does that mean?

The international body of human rights law, starting with the 1948 Universal Declaration of Human Rights, includes various provisions related to health and SDH. These include Article 25 of the Universal Declaration of Human Rights, Article 24(1) of the Convention on the Rights of the Child (1989/90), Article 5(e)(iv) of the Convention on the Elimination of All Forms of Racial Discrimination (1965/1969) and Articles 11(f) and 12 of the Convention on the Elimination of All Forms of Discrimination Against Women (1979/1981). Most notably, Article 12 of the International Covenant on Economic, Social and Cultural Rights proclaims "the right of everyone to the enjoyment of the highest attainable standard of physical and mental health," and obligates States Parties to ensure "provision for the reduction of the stillbirth-rate and of infant mortality and for the healthy development of the child; the improvement of all aspects of environmental and industrial hygiene; the prevention, treatment and control of epidemic, endemic, occupational and other diseases; and the creation of conditions which would assure to all medical service and medical attention in the event of sickness." (The United States has not ratified this Convention.) Although state obligations are limited to the progressive realization of the human right to health in the context of their "available resources" (Article 2), all states must show measurable progress towards its full realization. Assessing the extent of such progress requires evidence of effort to reach health goals, and of empirically grounded links between social and economic policy and health status trends within and between states.

In 2000 the UN Committee on Economic, Social and Cultural Rights issued General Comment 14 on Article 12, which both clarified the scope of the right to health and identified the obligations of states parties to respect, protect and fulfil the right [91]. General Comment 14 interpreted the right to health as an inclusive right that encompasses not only timely and appropriate health care, but also key underlying health determinants, including "access to safe and potable water and adequate sanitation, an adequate supply of safe food, nutrition and housing, healthy occupational and environmental conditions, and access to health-related education and information, including on sexual and reproductive health" (¶11). It further identified "core obligations" that include ensuring access to health facilities, goods and services on a non-discriminatory basis; to ensure access to minimum essential food and freedom from hunger; to ensure access to basic shelter, housing, sanitation and potable water; to provide essential drugs as defined by the World Health Organization Access Programme on Essential Drugs; and to adopt and implement a national public health strategy (¶43). It described the Article 12 obligations related to maternal and child health, industrial hygiene, and disease prevention and control as "obligations of comparable priority" (¶44). (For explication of Article 12 and General Comment 14, see [92-98].)

What would public policies that recognize health as a human right look like, and what might they mean for SDH? The question can usefully be considered in terms of potential impacts of trade policy on access to SDH. The United Nations' Special Rapporteurs on globalization and human rights concluded that "it is necessary to move away from approaches that are ad hoc and contingent" in ensuring that human rights are not compromised by trade liberalization [99](¶25). A more extensive inquiry was conducted by the Special Rapporteur on the Article 12 right to health (appointed in 2002, reappointed for a second term in 2005), whose first report adopted an expansive approach that links poverty reduction and the right to health [93]. A more recent report, dealing specifically with the WTO, found that "the progressive realization of the right to health and the immediate obligations to which it is subject, place reasonable conditions on the trade rules and policies that may be chosen" [94](¶24). Consequently, the report recommended *inter alia *"that urgent attention be given to the development of a methodology for right to health impact assessments in the context of trade" [94](¶74) – a challenge that is best viewed as part of the larger imperative of balancing the inherently commercial objectives of trade agreements with other social objectives such as poverty elimination [100].

WHO research has found that litigation to establish access to essential medicines as an actionable human right can succeed, mainly in situations where constitutional provisions entrench the right to health and/or acknowledge the primacy of international human rights agreements with respect to domestic policies and legislation [101]. The cases studied did not involve the provisions of trade agreements, although the intellectual property provisions of the Agreement on Trade-Related Aspects of Intellectual Property (TRIPs) remain central to debates about access to essential medicines despite a WTO interpretation that apparently offers flexibility with respect to compulsory licensing and parallel imports [102-107]. Neither did they address the more challenging question of how the right to health can be used to secure more equitable and widespread access to SDH such as adequate nutrition or safe water, which is specifically addressed in one of the MDG targets.

Indeed, the availability of safe water has often been reduced for the poor or otherwise vulnerable when costs rose as a consequence of privatization or the implementation of cost recovery measures [108-112]. Because it is essential to health, " [t]here are compelling arguments for viewing access to water as a human right," and water as a good whose commodification and commercialization should be limited [113](p. 567). On the other hand, this position is far from universally accepted; "the struggle persists because of reluctance among powerful players to acknowledge that principles of social and economic justice must not be sacrificed for reasons related to wider political economy" [113](p. 568). In another example with potentially far-reaching policy relevance, Hammonds and Ooms [97] have argued that many policies pursued by the World Bank, including expenditure ceilings and some aspects of loan conditionalities, lead to violations of member countries' obligations related to the right to health. How would this claim be adjudicated, and how would conclusions be implemented? These questions underscore the importance of a lack of implementation mechanisms – an issue that is unlikely soon to be resolved. Thus, the right to health as a counterweight to the priorities of the global marketplace offers important opportunities, but also formidable conceptual and practical challenges.

### The need to protect and expand "policy space"

"Policy space" has been defined as "freedom to choose the best mix of policies possible for achieving sustainable and equitable economic development given [developing countries'] unique and individual social, political, economic and environmental conditions" [114]. The concept is most often invoked with respect to how trade agreements constrain economic policy choices [100,115-117]. However, the effects of trade policy commitments on national policy space are not limited to those associated with the actual texts of trade agreements. For example, once such agreements have facilitated the reorganization of production across multiple national borders, governments' policy space is subsequently limited by the ability of a parent or lead firm to play off subsidiaries or independent contractors in multiple national jurisdictions against one another in order to minimize costs and maximize productivity. The effect of US retail giant Wal-Mart's procurement practices on suppliers in the developing world is sometimes cited as a case in point [118], but this is arguably just an especially conspicuous example of a dynamic and a concentration of power that is intrinsic to buyer-driven commodity chains [119-121].

Liberalization of financial markets enhances the power of the owners of financial assets relative to governments because of the (often implicit) threat of disinvestment. This process is familiar from the role of bond markets and credit rating agencies in defining the risks (to investors, not necessarily to residents of the country in question) associated with a particular government's policies, and therefore determining the interest rates bondholders will demand [122-124]. Fiscal discipline is also exercised in other ways; the implications for policies related to SDH can be understood starting from the premise that even when sustained economic growth is achieved, it cannot be assumed that gains from growth will be widely shared in ways that reduce poverty and other forms of vulnerability. Explicitly redistributive policies may be necessary.

As an illustration of this point, a recent study constructed alternative scenarios of progress by 18 Latin American and Caribbean countries – a region of the world where inequality is among the highest, on a variety of dimensions [125] – toward the MDG of reducing extreme poverty by 50 percent between 1990 and 2015. The study found "that even very small reductions in inequality can have very large positive impacts in terms of poverty reduction. For most countries considered, a one- or two-point reduction in the Gini coefficient," which is a standard measure of income inequality across an entire society, "would achieve the same reduction in the incidence of poverty as many years of positive economic growth" [126](p. 13). This represents an application at the country level of the New Economics Foundation's insight about the relative ineffectiveness of growth in reducing poverty worldwide, discussed in the second article of the series. In other words: *even a little economic redistribution could go a long way *toward reducing inequalities in access to SDH, especially if redistributive policies were combined with carefully designed publicly financed health system and educational interventions.

The nature of redistributive policies is that someone within the borders of the nation-state in question has to pay for them. The constraint on policy space that arises from the need to raise tax revenues to finance such measures, once again in the Latin American context, is succinctly described by Williamson, codifier of the "Washington consensus" on development policy [127] which throughout the 1990s focused on domestic deregulation and rapid integration of national economies into the global marketplace. " [I]t would not be practical," writes Williamson, "to push this very far, because too many of the Latin rich have the option of placing too many of their assets in Miami" [128]. The operation of this constraint is not limited to Latin America: financial deregulation and the increased mobility of financial assets have enabled the propertied worldwide to join "a sort of global, cross-border economic electorate, where the right to vote is predicated on the possibility of registering capital" [129](p. 40).

Evidence that interjurisdictional competition has already reduced fiscal capacity and constrained the ability of governments to increase the progressivity of taxation and improve the effectiveness of tax collection is inconclusive [130-132]. The former Chief of the IMF's Public Finance Division has predicted that this constraint will clearly arise in the future [133]; he has identified several "fiscal termites" including inability to tax financial capital, accounting flexibilities associated with intrafirm trade across national borders, the proliferation of derivatives and hedge funds, and the cross-border mobility of high income earners [134] that will limit fiscal capacity and start chewing on the foundations of tax systems in countries rich and poor alike (see also [135,136]). For some observers, the ideal remedy would be multilateral agreement on the creation of a system for global taxation and redistribution of resources across national borders, such as the long-standing proposal for a tax on currency transactions – the "Tobin tax" – or more recent proposals for taxes on carbon emissions or air travel [137-141]. France has now adopted a tax on air tickets, the progressivity of which is maintained by a much higher tax on business class tickets, with proceeds dedicated to supporting purchase of drugs to treat AIDS, tuberculosis and malaria in developing countries [142]; 13 other countries have signed on to this proposal [143]. It remains to be seen whether the existence of this levy will create a political obstacle to increasing development assistance from general revenues in the industrialized world, and such purpose-specific funds are no substitute for the larger scale global redistribution that some would argue is ethically imperative.

This necessarily brief discussion suggests a rather bleak conclusion. Redistributive policies of various kinds are likely to be needed to reduce health inequities within and between countries. Globalization tends to be associated with a long-term trend toward increasing economic inequality and increasing attachment to markets as a mechanism for allocating resources and setting policy priorities. At the same time, globalization generates constraints on the ability of national and sub-national governments to implement the policies that would mitigate or compensate for those impacts. Identifying 'success stories' of effective interventions is therefore especially important. However, it must to be asked whether the interventions in question are genuinely improving health equity, or are simply undoing some of the damage done by integration into the global marketplace, and how sustainable they are in view of pressures toward (for example) labour market flexibility and tax competitiveness.

Because of the limited universe of case studies (how many governments in the world have actively and aggressively been concerned with reducing health inequity?) and the associated need to rely on counterfactuals (what would have happened if they had been more concerned?), much is not known about the policy space for measures to reduce health equity by way of addressing the social determinants of health. Scenario construction and analysis may be the best way of reducing this knowledge gap, and it is a central element in a multi-year team project on globalization and health disparities in major Canadian metropolitan areas that is now getting under way. The authors are respectively principal investigator and co-investigator on this study, which involves an additional 18 co-investigators from a total of 10 universities, as well as a number of collaborators and consultants from civil society organizations.

### Conclusion: SDH and values for the global community

Jeffrey Sachs has noted that "in a world of trillions of dollars of income every year, the amount of money that you need to address the health crises is easily available in the world" [144](p. 3). Scarcity of resources, in any absolute sense, is not the issue. Rather, the issue is one of whether and how resources necessary to meet the basic needs of the world's majority will be mobilized rapidly and effectively.

Studying the key elements of contemporary globalization leads one to contrast two fundamentally distinct visions of the future, which often are only implicit in policy discussions and are presented here in stylized form.

In the first vision, individuals, households, and national economies have to 'earn their keep' in the global marketplace. This offers major opportunities for some, and major risks – exemplified by long-term unemployment, economic insecurity and marginalization, and catastrophic illness – for others. This vision does not preclude social policy interventions, but they must be justified in terms of the return on investment. Investing in health (the mantra of the Commission on Macroeconomics and Health) is defended with reference to evidence of the payoffs in improving the ability of individuals, households and societies to compete in the global marketplace. The triages that are implicitly accepted in the vocabulary of investing in health in developing countries have received too little attention from development and population health researchers.

More broadly, this first vision redefines social protection as "social risk management," in the words of a World Bank strategy document advocating "a new conceptualization of social protection that is better aligned with current worldwide realities" [145](p. 1, 9). The initial presumption is that " [i]n an ideal world with perfectly symmetrical information and complete, well-functioning markets, all risk management arrangements can and should be market-based (except for the incapacitated) [145](p. 16). The fundamental task of social policy is redefined in radically individualistic terms, as helping households "to smooth their consumption patterns" in response to exogenous events ranging from natural disasters to financial crises [145] (p. vii-ix). Governmental intervention to help the non-incapacitated poor is justified only when "market failures" result from the fact that the poor "are more vulnerable than other population groups because they are typically more exposed to risk and have little access to appropriate risk management instruments" [145] (p 10). Because the norms of the market are taken as given, no attention is paid either to normative considerations of social justice or to the empirical question of how (for example) promotion of trade liberalization and financial integration has facilitated capital mobility in search of lower production costs, thereby allowing investors to *create *the "worldwide realities" that are invoked to justify a new generation of domestic social and economic policies to integrate people and countries into the global marketplace.

The second vision seeks to blunt the negative impact of the emerging global marketplace. This vision, which lacks codifiers as authoritative and well financed as institutions like the World Bank, incorporates such perspectives as:

o Institutionalized recognition of at least minimal access to the material prerequisites for health as a human right, with corollary claims against available resources;

o the call of the International Labour Office's World Commission on the Social Dimensions of Globalization for a new form of globalization that recognizes social obligations and incorporates new institutions for global governance [146];

o the reference by international relations and human rights scholar Richard Falk [147] to "a regulatory framework for global market forces that is people-centred rather than capital-driven" (p. 18);

o the invocation by Michael Marmot, now Chair of the Commission on Social Determinants of Health, of "public policy based on a vision of the world where people matter and social justice is paramount" [148](p. 1099); and

o despite its conceptual shortcomings, the idea of a "global social contract" analogous to the social contract within industrialized countries that supports contemporary welfare states [16].

The formidable barriers to implementing the second vision we have identified can be understood, in part, by unpacking the social contract analogy. A long and sometimes violent history of political conflicts (notably, but not exclusively, between capital and labour) preceded the implicit contract that underpins many contemporary welfare states and legal frameworks for industrial relations. Those conflicts were resolved, and the implicit contracts forged, in a context where national boundaries largely defined the options available to all parties. Today, given the shifts of bargaining power that have accompanied the emergence of contemporary globalization, it is not certain that (for instance) investors with the option of capital flight or the managers of transnational corporations would see the need for such contracts ... and they are meanwhile unravelling domestically in many high-income welfare states, especially Anglo-American ones (see e.g. [149,150]).

Another set of barriers to implementation in the context of trade policy has been identified by Stiglitz & Charlton [18], who noted that living up to the rhetorical identification of the multilateral negotiations that began in 2001 as a "development round" was likely to require "a fundamental departure from the system of mercantilism," driven by considerations of national interest or by the economic interests of particularly powerful economic actors within nations (p. 496). The apparent collapse of WTO negotiations in July 2006 underscored the magnitude of that departure, and the generic difficulty in using what is essentially an ethical argument in matters of international relations. However, the alternative to the use of such arguments in support of economic, social and foreign policies that are conducive to health equity and improving the social determinants of health is the implied position that growth through marketization will benefit everyone in the long term and whatever health damage occurs in the interim must be accepted as the price of progress. Stated in this manner, the position makes consideration of issues of distributive justice unavoidable – hence, returning us to the concept of health equity with which the series of articles began.

This discussion suggests a provocative parallel: in both trade policy and human rights, institutions and norms of global governance have emerged. A crucial difference between the two is that no multilaterally agreed upon implementation and enforcement mechanisms exist with respect to human rights that are even roughly comparable to dispute resolution procedures under trade agreements. Exploring the political possibilities for developing such mechanisms organized around the right to health, or some alternative concept that embodies a comparable challenge to the norms of the global marketplace, would require an additional article (or series of articles). The task remains urgent, and we conclude by quoting an axiom about contemporary globalization that applies among as well as within nations: "At the very least ... those who stand to benefit from the process should be expected to agree to provide systematic and substantial assistance to the victims, presumably via government channels, and supported liberally by the wealthier communities. If that is not acceptable politically, there is surely little that can be said convincingly in support of a contention that the suffering of the victims will be justified by the promised future benefits to their descendants" [151] (p. 430).

## Competing interests

The author(s) declare that they have no competing interests.

## Authors' contributions

The authors contributed equally to the conception and design of the study; acquisition, analysis and interpretation of data; and drafting of the manuscript. Both authors have read and approved the final manuscript.
